# Beyond Survival: Assessing Quality of Life, Activity Level, and Ethnic Diversity in Aortic Dissection Survivors

**DOI:** 10.3390/jcdd12020078

**Published:** 2025-02-18

**Authors:** Nora Bacour, Olivia van Erp, Simran Grewal, Aytug U. Tirpan, Susanne Eberl, Kak Khee Yeung, Ron Balm, Germaine C. Verwoert, Antoine H. G. Driessen, Robert J. M. Klautz, Nimrat Grewal

**Affiliations:** 1 Department of Cardiothoracic Surgery, Amsterdam University Medical Center Location AMC, 1105 AZ Amsterdam, The Netherlands; n.bacour@amsterdamumc.nl (N.B.); a.u.tirpan@amsterdamumc.nl (A.U.T.); a.h.driessen@amsterdamumc.nl (A.H.G.D.); r.klautz@amsterdamumc.nl (R.J.M.K.); 2 Shoulder and Elbow Unit, Department of Orthopaedic Surgery, Onze Lieve Vrouwe Gasthuis, 1091 AC Amsterdam, The Netherlands; s.k.grewal@olvg.nl; 3 Department of Anesthesiology, Amsterdam University Medical Center Location AMC, 1105 AZ Amsterdam, The Netherlands; s.eberl@amsterdamumc.nl; 4 Department of Vascular Surgery, Amsterdam University Medical Center, Location University of Amsterdam, 1105 AZ Amsterdam, The Netherlands; k.yeung@amsterdamumc.nl (K.K.Y.); r.balm@amsterdamumc.nl (R.B.); 5 Department of Vascular Surgery, Amsterdam Cardiovascular Sciences, Amsterdam University Medical Center, Location Vrije Universiteit Amsterdam, 1081 HV Amsterdam, The Netherlands; 6 Department of Cardiology, Basalt, 2333 AL Leiden, The Netherlands; g.verwoert@basaltrevalidatie.nl; 7 Department of Cardiothoracic Surgery, Leiden University Medical Center, 2333 ZA Leiden, The Netherlands; 8 Department of Anatomy and Embryology, Leiden University Medical Center, 2333 ZA, Leiden, The Netherlands

**Keywords:** acute aortic syndrome, aortic dissection, quality of life, activity, survivors

## Abstract

Background: An acute aortic dissection (AAD) is a highly lethal condition that demands immediate medical intervention. Survivors often face significant long-term challenges. While immediate survival remains a critical focus in acute care settings, little is known about long-term results, especially with regard to activity levels, post-operative quality of life, and the impact of cultural and ethnic characteristics on recovery. Using data from the Dutch National Aortic Dissection Survivor’s Day, this study examines QoL, activity levels, and ethnic diversity among survivors. Methods: All patients (*n* = 45) participating in a national awareness meeting for AAD survivors at a teaching hospital were included in our study. Participants completed questionnaires assessing QoL, activity levels, and sociodemographic data, including ethnic background. The EQ-5D and IPAQ surveys were employed to measure health-related QoL and physical activity, respectively. Results: The mean age of the participants was 59.5 years, with 37.8% identifying as women. Ethnically, 88.9% identified as Dutch. The majority reported good health (mean score of 73/100). However, there was a considerable variation in QoL scores. On average, 22.2% of our study population reported moderate or greater problems across all dimensions, compared to 5.5% in the normative sample. Activity levels were mostly low to moderate, and no significant differences in QoL were found based on activity levels. Sleep quality was generally good. Conclusions: Our study reveals significant limitations in QoL among AAD survivors. This emphasizes the significance of developing a multimodal rehabilitation program focused on addressing current gaps in recovery. Ultimately, this will enhance overall care following AAD. Future research should focus on assessing long-term QoL.

## 1. Introduction

An acute aortic dissection (AAD) is a rare condition with high lethality that demands immediate medical intervention [1]. Advances in disease awareness, medical diagnostics, and timely surgical management have significantly improved the survival rates of type A AAD, from less than 40% to over 80% [2,3]. Although surviving an AAD is an important milestone, it often marks the start of a complicated and extensive recovery journey, with many patients forced to adjust to long-term psychological and physical difficulties. While immediate survival remains a critical focus in acute care settings, long-term outcomes, particularly in the domain of quality of life (QoL), remain underexplored.

Limited available literature suggests that survivors may continue to face numerous challenges, including postoperative complications, reduced physical function, psychological trauma, and diminished QoL that tends to continuously decline over time [4,5]. Furthermore, the role of ethnicity and cultural factors in shaping patients’ experiences and outcomes has often been neglected in the literature [6,7]. This is a concerning gap in current knowledge, as evidence accumulates that ethnic minority patients often encounter barriers to equitable medical care, such as cultural miscommunication and inadequate health literacy [8]. Breel et al. highlighted in their paper that patients recovering from an AAD often expressed the need for improved post-discharge guidance and communication [9].

In the Netherlands, national efforts to engage AAD survivors through events such as the National Aortic Dissection Survivor’s Day provide a unique opportunity to capture patient-reported outcomes and experiences. These gatherings not only foster peer support but also serve as a platform to gather insights into the lived experiences of survivors, including their physical activity level, psychosocial well-being, and perception of care.

This study aims to fill critical gaps in understanding post-dissection recovery by describing the QoL, activity levels, and ethnic diversity among survivors of an AAD. By leveraging patient-reported data collected during a national event, we seek to explore how ethnicity and cultural factors intersect with physical and psychological recovery, and how these insights can inform more equitable and personalized rehabilitation strategies.

## 2. Materials and Methods

### 2.1. Ethical Approval

The study protocol was reviewed and approved by the Medical Ethical Committee of Leiden University Medical Center under the biobank protocol with reference number: B21.051/MS/ms. All participants were asked to sign informed consent forms before participating in the study. The Declaration of Helsinki’s ethical principles and the norms for good clinical practice were followed, guaranteeing the privacy and confidentiality of all information gathered.

### 2.2. Patient Selection

This study was conducted using questionnaires distributed during a national event for AAD survivors, held in September 2024 at the Amsterdam UMC, a teaching hospital in Amsterdam, the Netherlands. A target population of 45 patients was anticipated. During the event, QR codes linking to digital questionnaires were provided to the participants, allowing AAD survivors to complete the survey. Survey answers were obtained from all participants attending the national event.

### 2.3. Questionnaires

#### Sociodemographic Questionnaire

Demographic variables included age, sex, weight, height, presence of risk factors (e.g., smoking), level of education, employment status, ethnicity, and nationality. Ethnicity was self-reported with options being Dutch or other, using an open-ended text form. Education level was categorized as low (primary education), moderate (secondary education), or high (higher professional or university education), and employment status was defined as working, retired, disabled, or other.

### 2.4. Health Survey

To supplement sociodemographic data with a thorough assessment of QoL and activity levels, we used established, validated questionnaires. The IPAQ-SF survey [10] was used to evaluate post-operative physical activity levels and to explore potential ethnic differences in recovery and activity. The responses categorized participants’ activity levels as low, moderate, or high based on predetermined scoring criteria [11]. A high level of physical activity refers to daily moderate intense physical activity for an hour or more. Moderate level of physical activity refers to activities that are equivalent to 30 min of moderately intense physical activity on most days. Low level of physical activity refers to participants who do not meet the requirements for either moderate or high levels of physical activity [11].

Health-related QoL was measured using the EQ-5D-5L questionnaire [12]. The EQ-5D-5L survey measures health-related QoL across 5 dimensions: mobility, self-care, usual activities, pain/discomfort, and anxiety/depression. Each dimension received a score ranging from 0 and 4, where 0 indicated no problems and 4 indicated extreme problems in the represented field. These scores were compared to population norms from a European country, as Dutch norms were not available [13]. Additionally, the responses were used to calculate the average QoL using a key from the Dutch EQ-5D-5L value set [14]. This value set enabled scoring of overall QoL, the EQ-index, on a scale ranging from −1 to 1, where 0 represents being equivalent to death. Scores below 0 indicate a QoL perceived as worse than being dead, with values closer to −1 reflecting increasingly poorer QoL. Conversely, positive scores represent better QoL, with higher values indicating more optimal outcomes.

Furthermore, a scale from 0 to 4 was used to assess sleep quality, with 0 indicating no problems and 4 indicating extreme issues. This was further supplemented by average sleep duration. Patients were further asked what type of AAD they had (type A, with or without extension into the descending aorta, or type B) and whether they underwent a surgical intervention or conservative treatment.

Patients were asked to score their perceived health on a scale of 0 to 100, where 100 represented the best state of health. Patients were also asked to assign a score to their perceived care on a scale from one to ten, where zero represented the worst possible care and ten represented perfect care. To investigate ethnic inequalities, we asked patients about their healthcare perceptions and whether they understood the information provided. They were also asked to assess the quality of received educational materials and care services.

### 2.5. Statistical Analyses

The baseline characteristics and survey responses of the post-dissection patients were summarized using descriptive statistics. Descriptive continuous data are displayed as mean ± standard deviation, whereas categorical data are displayed as frequencies and percentages. SPSS-29 software was used to further analyze the data. Firstly, data were analyzed in terms of skewness. Disparities between the two groups were then examined in different manners.

Continuous normally distributed data were assessed using an unpaired *t*-test. Continuous non-normally distributed data were examined using a Mann–Whitney U test. For categorical data, a chi-square test was employed (normal or for trend). If cell counts of the chi-square were insufficient, the data were instead analyzed using a Mann–Whitney U test. Disparities between the three groups were assessed using a one-way ANOVA for normally distributed data and a Kruskal–Wallis test for non-normally distributed data. *p*-values below 0.05 were considered statistically significant.

## 3. Results

### 3.1. Baseline Demographics

Our study population included 45 participants, with a mean age of 59.5 years (SD ± 8.5). The sample consisted of 37.8% women and 62.2% men. Participants had an average weight of 84.9 kg (SD ± 15.9 kg) and an average height of 180 cm (SD ± 9.3 cm). According to reported smoking habits, 46.7% of people had formerly smoked, whilst 2.2% were current smokers. The majority of participants, 55.6%, were diagnosed with Type A AAD. Patients reported an average health scale score of 73 out of 100 (Table 1).

Regarding treatment, 91.1% of participants underwent surgical intervention, while 8.9% were managed conservatively. Among the study population, the majority, 27 participants (60.0%), had a high degree of education, and nearly half, with 22 participants (48.9%), were still employed. Sleep quality was rated as either fairly good or very good by 75.6% of participants, with an average sleep duration of 6.9 h (SD ± 0.9) (Table 1).

In terms of information and care, 31.4% of participants reported difficulties in understanding their care due to issues such as a lack of tailored information, delayed communication, and insufficient details. Additionally, 17.1% found the informational materials unhelpful. The average rating for care services was 7.5 on a scale of 10 (SD ± 1.2), with 53.3% of patients rating their received healthcare as ‘good’.

### 3.2. Ethnicity

Ethnically, the majority (88.9%) identified as Dutch, while 11.1% reported being of ethnic descent, with one participant of Turkish origin (2.2%), two from the Dutch Antilles (4.4%), and two of mixed origin (Dutch/Congolese and Dutch/Dutch Antillean) (4.4%). Notably, one participant of Dutch/Congolese origin expressed feelings of not being taken seriously regarding their complaints, attributing this to their ethnicity. The low number and heterogeneity of ethnic groups posed challenges in accurately assessing individual disparities related to ethnicity in care evaluations. When comparing the average perceived healthcare scores, the non-Dutch ethnic group non-significantly reported slightly lower average scores compared to the Dutch ethnic group (7.25 vs. 7.52; *p* = 0.694).

### 3.3. Quality of Life

**Figure 1:**
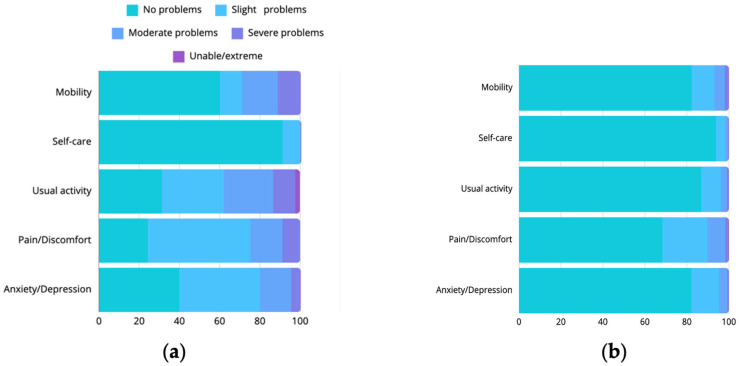
This figure shows the quality of life assessment in our study (**a**), compared to average EQ-5D dimensions (**b**) [15]. It shows a decline in QoL across all aspects in post-dissection patients. This underscores the importance of implementing multimodal recovery programs to enhance QoL.

**Figure 2:**
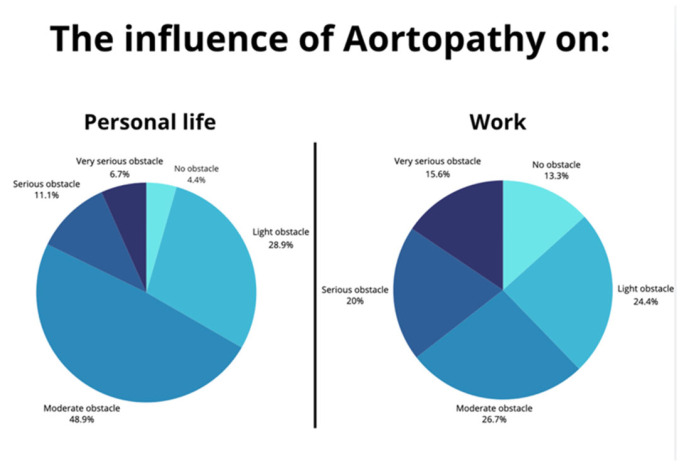
Impact of aortopathy on daily life and work. This figure shows influence of Aortopathy on personal life and work among AAD survivors. It is evident that only a small percentage of patients experience no impact on their work or personal life due to aortopathy. This highlights the importance of providing high-quality care to address these challenges and improve life after dissection.

The impact of aortic disease on daily life varied among the participants. QoL was assessed in terms of mobility, self-care, usual activities, pain/discomfort, and anxiety/depression (Figure 1). The impact of aortic disease on daily life varied across patients, with the biggest group reporting a moderate impact on both work (26.7%), and personal life (48.9%) (Figure 2). Compared to participants with a Type A dissection, participants with a Type B dissection experienced significantly greater limitations in mobility (*p* = 0.024), self-care (*p* < 0.00), and pain (*p* = 0.011), as well as non-significantly with anxiety (*p* = 0.194) or daily activities (*p* = 0.154). Regarding enthusiasm for performing daily tasks, 15.9% reported no issues, while 40.9% experienced slight, 34.1% moderate, and 9.1% major problems. Higher rates of reported difficulties in anxiety (*p* = 0.001), pain (*p* = 0.023), daily activities (*p* = 0.001), and mobility (*p* = 0.024) were found to be associated with lower reporting of enthusiasm for daily tasks. Figure 2 presents the QoL assessment from our study in comparison to the average EQ-5D dimension of the general population in Germany [15]. On average, 22.2% of our study population reported moderate or greater problems across all dimensions, compared to 5.5% in the normative sample. The overall EQ-index averaged 0.7 (SD ± 0.2), which is lower compared to the average of 0.9 reported in the Dutch value set [14].

### 3.4. Activity Levels

Participants engaged in heavy training for an average of 0.9 days per week for 15.2 min per session and moderate-intensity training for 1.5 days per week for 41.6 min per session. They walked an average of 40.1 min per session during an average of 3.6 days a week and spent approximately 6 h and 13 min sitting or lying down on all days. Lastly, activity levels as assessed by the International Physical Activity Questionnaire (IPAQ) [16], revealed that most patients were “low” in activity (40.5%), though a smaller group was moderately (38.1%) or highly active (21.4%). The mean, however, showed that participants were moderately active. The average EQ-index did not significantly differ based on activity levels (low 0.6 vs. moderate 0.5 vs. high 0.8; *p* = 0.052).

## 4. Discussion

In this study, we aimed to assess the QoL, activity levels, and ethnic diversity in survivors of aortic dissection.

### 4.1. Ethnicity

Our study population predominantly consisted of Dutch and Caucasian participants, with only 11.1% identifying as part of an ethnic minority. Concurrently, 25.4% of the Dutch population reports a non-Dutch ethnic background [17]. This under-representation reflects the broader issue in medical research that ethnic minorities tend to be insufficiently represented in medical research [18]. Patients from ethnic backgrounds may be less likely to participate in community events because they are less fluent in the language. An additional hypothesis is that different ethnic groups have different chances of having an AAD. This notion is supported by two studies conducted in New Zealand that show differences in the incidence of acute aortic syndromes (AAS), with Pacific Islander and Indigenous Māori patients having a higher frequency of AAS than other ethnic groups [19,20]. However, the contribution of socioeconomic factors, access to preventive care, or other underlying causes to this disparity remains unclear [19]. Future research should strive to these hypotheses and examine the likelihood of AAD across ethnic groups in larger studies.

The slightly lower overall care assessment scores between the Dutch and non-Dutch ethnic groups, combined with the Dutch–Congolese participant’s experience of not feeling taken seriously by medical professionals, raise questions about possible gaps in care for ethnic minorities. These disparities may be related to inadequate information provision and communication barriers.

Ethnic minority groups often face struggles in terms of language and cultural barriers, as well as negative societal discourses [21,22]. Further research should focus on the unique cultural and psychological difficulties and subjective needs that make for better post-operative support. Educating healthcare professionals on cultural competency becomes imperative to enhance intercultural care and reduce perceived inequities [23]. Taken together, creating tailored, culturally attuned healthcare information and improved cultural knowledge and communication strategies, could positively impact recovery outcomes for all patients, but specifically for ethnic minorities.

### 4.2. Quality of Life and Activity Levels

Survivors of acute aortic dissection in our study exhibited a wide range of QoL and activity levels, reflecting the complexity of their recovery processes. Although most participants rated their health as ‘good’, many reported significant limitations in their QoL, particularly in terms of mobility and daily activities. We compared the QoL assessment to the average EQ-5D dimension of the general population of a neighboring country (Germany [15]), as the normative values from the Netherlands are lacking. This comparison showed a lower QoL in aortic dissection survivors, which is consistent with previous research [24].

While the average patient was classified as moderately active according to the International Physical Activity Questionnaire (IPAQ) [16], their activity levels did not significantly influence the average reported QoL. Moreover, studies show that AAD survivors experience alterations in lifestyle and emotional state, with high rates of new-onset depression (32%) and anxiety (32%) after a dissection [25]. These findings imply that physical recovery alone may not be sufficient; psychological factors also play a crucial role. According to research conducted by Remskar et al. [26], interventions that combine psychological support with physical rehabilitation have shown promise in enhancing both mental and physical health. Furthermore, research by Lang et al. [4] highlights the value of organized post-surgical rehabilitation programs that emphasize social support, education, and self-regulation—all of which have been connected to improved recovery results. Thus, interventions that encompass both physical and mental recovery are vital for enhancing QoL and activity levels among survivors of AAD.

### 4.3. Limitations

Recognizing the limitations of our research is essential. The low number of Type B dissection patients and the low participation rate of ethnic minorities within our study population pose limitations to identifying potential discrepancies in these fields. This limitation also offers a chance to highlight the need for future research to look for methods to incorporate a wider variety of patient populations. This will advance our understanding of how ethnicity influences the recovery process after AAD and disparities in needs when comparing different types of aortic dissections. In turn, this may facilitate the development of interventions that are specifically designed to match the needs of each survivor.

Another important limitation is our patient selection. Participants in the national awareness meeting are members of the National Dissection Patient Association, often motivated individuals. These participants might have a higher level of engagement compared to the average dissection patient. Additionally, participants who attended must have been able to understand the Dutch invitation, creating a selected group of individuals proficient in the language. This is particularly relevant for ethnic subgroups, who may face greater challenges in understanding healthcare information and may not have been able to attend the gathering. While a larger cohort would be ideal, studies on rare conditions often rely on smaller sample sizes, and our sample size is comparable to those in previous research on dissection survivors [9,27].

Lastly, while our study describes life after an AAD across various domains, it does not explore its potential association with clinical parameters. This may limit the understanding of the reasons or risk factors behind a deteriorating recovery process. However, descriptive studies serve as an essential first step in identifying trends and generating hypotheses for future research. Particularly in rare conditions such as thoracic aortopathy, descriptive data are crucial for conducting future, more detailed investigations.

## 5. Conclusions

In conclusions, our findings highlight variability in QoL, which emphasizes the importance of integrating both physical and psychological rehabilitation. Moreover, our study exposes gaps in understanding the influence of ethnicity on the recovery process. This underscores the urgent need for further exploration of how cultural perspectives and health literacy shape postoperative experiences.

Future research should prioritize longitudinal studies assessing the long-term QoL of AAD survivors while at the same time studying ethnic disparities in the prevalence of AAD in larger studies. Concurrently, it is essential to explore ways to improve rehabilitation programs. Promoting greater cultural awareness is an important area for improvement, particularly given the high anxiety levels among ethnic minorities in our study. By developing culturally sensitive interventions, we can improve post-dissection recovery while ensuring that care for AAD survivors is both effective and efficient, ultimately improving the quality of life for all patients.

## Figures and Tables

**Table 1:** Patient characteristics.

	*n* (45)	Mean	Minimum	Maximum	Std. Deviation
Age	37 (8 missing)	59.5	30.5	73.3	8.5
Sex	17 (37.8%) Female 28 (62.2%) Male				
Weight (kg)	45	84.9	60	155	15.9
Height (cm)	44	180	158	193	9.3
Type of dissection					
Type A dissection	25 (55.6%)				
Type B dissection	4 (8.9%)				
Tyle A dissection with extension into the descending aorta	14 (31.1%)				
Missing data	2 (4.4%)				
Surgical intervention	41 (91.1%)				
Dutch ethnicity	40 (88.9%)				
Smokers	1 current (2.2.%) 21 ex-smokers (46.7%) 23 non-smokers (51.1%)				
Average hours of sleep	45	6.9	3	10	0.9
Educational level	27 (60%) High 16 (35.6%) Middle 2 (4.4%) Low				
Employment status	22(48.9%)working 7 (15.6%) retired 12 (26.7%) incapacity 4 (8.9%) other				
Health scale rating	45	73	40	95	15.2
EQ-index	45	0.7	−0.1	1.0	0.2

## Data Availability

The datasets generated for this study can be made available upon reasonable request.

## Abbreviations

The following abbreviations are used in this manuscript:

AAD	Acute Aortic Dissection
SADN	Stichting Aorta Dissectie Nederland (Dutch Aortic Dissection Foundation)
QoL	Quality of Life
